# Outcomes following elective cerclage versus ultrasound surveillance in women with one prior preterm event

**DOI:** 10.1016/j.ejogrb.2023.09.001

**Published:** 2023-11

**Authors:** Joshua Mullin, Hannah Rosen O'Sullivan, Andrew H. Shennan, Natalie Suff

**Affiliations:** Department of Women and Children's Health, School of Life Course and Population Sciences, Faculty of Life Sciences and Medicine, King's College London, London, UK

**Keywords:** Preterm birth, Elective cerclage, Ultrasound-indicated cerclage, Cervical cerclage, Mid-trimester loss

## Abstract

**Objective:**

Preterm birth, defined as delivery before 37 weeks’ gestation, is a major obstetric challenge and is associated with serious long-term complications in those infants that survive. Preventative management includes cervical cerclage, either as an elective procedure or performed following transvaginal ultrasound surveillance and shortening of the cervix (≤25 mm). Significant questions remain regarding the optimal management, target population and technique. Therefore, this study aimed to assess differences in risk factors and pregnancy outcomes for women who received an elective cerclage versus ultrasound surveillance, following one prior premature event (spontaneous preterm birth/second trimester loss).

**Study design:**

Women were retrospectively identified from St Thomas’s Hospital Preterm Birth Clinical Network Database. Women who had one prior premature event (between 14^+0^ and 36^+6^ weeks’ gestation) were included and they were separated into those that an elective cerclage and those who underwent ultrasound surveillance to assess differences in demographics, pregnancy risk factors and preterm birth outcomes. We excluded women who received other preventative therapies. We also separately analysed those women who required an ultrasound-indicated cerclage, comparing the differences between women that delivered preterm and term.

**Results:**

We collected data from 1077 women who had a prior preterm event. 66 women received an elective cerclage. 11.4% of women who had ultrasound surveillance received an ultrasound indicated cerclage. Women with a prior history of mid-trimester loss, instead of preterm birth, were more likely to receive an elective cerclage. The mean gestational age of delivery was similar between those women who received an elective cerclage and those who had ultrasound surveillance with and without an ultrasound-indicated cerclage (38^+1^ vs 37^+1^), however, preterm birth rates <37 weeks’ were twice as high in this ultrasound group (OR 2.3 [1.1–4.5], p = 0.02). In those women that do require an ultrasound-indicated cerclage, 50.4% deliver preterm.

**Conclusions:**

In conclusion, this study shows that in women with one prior preterm event, both history-indicated cerclage and ultrasound surveillance are appropriate management options. The majority of women undergoing ultrasound surveillance did not require a cerclage and so avoided the potential perioperative complications of cerclage insertion. However, those that did require an ultrasound-indicated cerclage were at high risk of preterm birth so should be followed up closely to enable adequate preterm birth preparation. Further prospective studies comparing history indicated cerclage and US surveillance in women with one prior preterm event are necessary.

## Introduction

Preterm birth, defined as delivery before 37 weeks’ gestation, leads to an estimated 15 million babies born early each year worldwide [1]. In the UK around 8% of births are preterm, which equates to around 60,000 infants each year [2]. Clinical outcome is inversely correlated to gestational age at delivery, with babies born before 28 gestational weeks at the highest risk of mortality and morbidity [3]. Current preventative management of women at high risk of preterm delivery includes the use of a cervical cerclage to provide mechanical support to the cervix and its mucus plug during pregnancy.

Transvaginal cerclages can vary by indication. Firstly, they can be indicated by a woman’s history, due to risk factors for preterm birth, and placed electively from 10 weeks’ gestation. Alternatively, they can be indicated by ultrasound surveillance, shown by a shortening of the cervix (<25 mm in length) and typically placed any time up to 24 weeks’ gestation. The RCOG guidance recommends that an ultrasound-indicated cerclage should be offered to women with a cervical length <25 mm if they have had one or more spontaneous preterm births (sPTB) and/or mid-trimester losses (MTL), whilst a history-indicated or elective cerclage is only recommended following three preterm events or losses [4], [5]. However, in practice and in the age of the Montgomery test where patient informed choice is paramount, clinicians often insert elective cerclages following one preterm event [6], [7].

This study aimed to assess the management of women seen in the premature surveillance clinic following one prior preterm event (sPTB/MTL). We specifically compare outcomes in women who have had an elective cerclage, with those who have ultrasound surveillance with a subsequent ultrasound-indicated cerclage if indicated.

## Methods

This is a retrospective cohort study conducted at St Thomas’s Hospital, London UK. The study had ethical approval under the Preterm Birth Clinical Network Database (Research Ethics Committee (REC) Reference 16/ES/0093) [8]. Women who attended the Preterm Surveillance clinic between 1st November 2012 and 1st August 2022 were identified from the database. Pregnant women were eligible for inclusion if they accessed care at the preterm surveillance clinic, had a history of a single spontaneous preterm birth or mid-trimester loss (between 14^+0^ and 36^+6^ weeks), and were currently pregnant with a singleton. Women were excluded if they received a transabdominal cerclage, progesterone or an arabin pessary for the purpose of preterm birth prevention, had a fetus with a known congenital abnormality, presented with vaginal bleeding or rupture of fetal membranes. An emergency rescue cerclage was defined as a cerclage performed when the fetal membranes were exposed on ultrasound or speculum examination with no clinical evidence of chorioamnionitis (including maternal fever, raised white blood cells and raised c-reactive protein).

After identification of eligible women, data was collected from patient notes, from our online maternity documentation system, Badgernet® (Clevermed) and directly from the Preterm Birth Clinical Network Database. Demographic data was obtained including age, BMI, ethnicity, as well as smoking status and preterm birth risk factors. The primary outcome was gestation at delivery. Secondary outcomes included patient demographics, delivery <32 weeks’ gestation, delivery <24 weeks’ gestation and additional preterm birth risk factors, as well as labour and neonatal outcomes (including birth weight, neonatal death, admission to neonatal unit).

Statistical analyses were performed using GraphPad Prism version 9.0 (GraphPad, San Diego, CA). Non-parametric continuous data was analysed using Mann-Whitney rank tests. Categorical data was analysed using Fisher’s exact test. P < 0.05 was considered to be statistically significant.

## Results

**Fig. 1:**
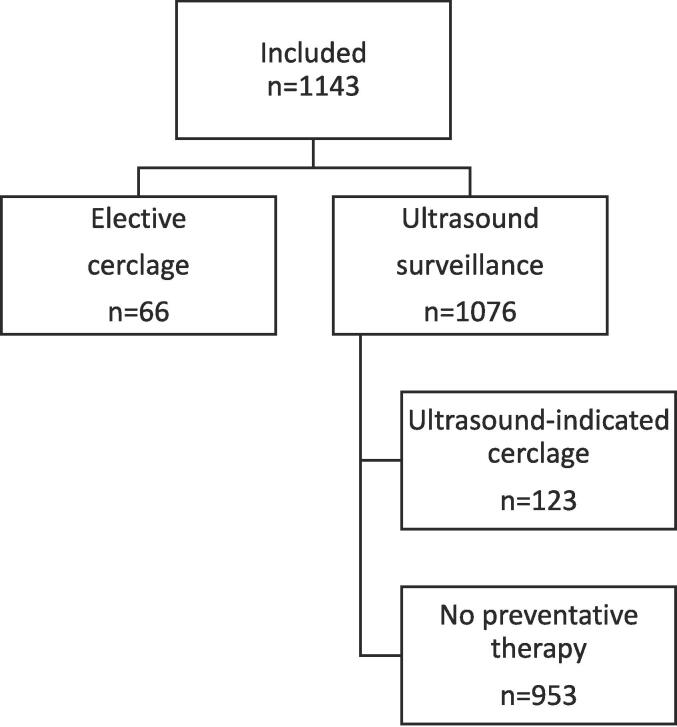
Flowchart of patients included in the study.

1143 women were seen in our Preterm Surveillance clinic who had one prior preterm event (sPTB/MTL) over the study period (Fig. 1). A total of 1077 woman had ultrasound surveillance with 123 women requiring an ultrasound-indicated cerclage due to cervical shortening; this equated to 11.4% of women. 66 women had an elective cerclage, which was arranged following their first clinic appointment.

The demographics were similar between the elective cerclage and ultrasound surveillance groups (Table 1). There was, however, a higher proportion of women with a prior MTL in the elective group (elective; 62.2% vs ultrasound; 44.2%, p = 0.004), whilst there was a higher proportion of women with a prior sPTB in the ultrasound surveillance group (elective;37.8% vs ultrasound;55.8%, p = 0.004). The difference in the gestation of the prior PTB event between the 2 groups was 24 weeks’ gestation in the elective cerclage group, compared to 32 weeks’ gestation in the ultrasound surveillance group. Additional risk factors for preterm birth, including cervical surgery, were similar between the 2 groups (Table 1).

**Table 1:** Demographics comparing women with history-indicated cerclage and ultrasound surveillance.

	Elective cerclage (n = 66)	Ultrasound surveillance (n = 1076)
**Age (mean)**	34.0	33.4
**BMI (mean)**	27.0	27.0

**Ethnicity % (n)**		
European	44 (29)	38.3 (413)
African	27 (18)	32.5 (350)
Afro-Caribbean	11 (7)	12.7 (137)
South Asian	6 (4)	3.5 (38)
East Asian	6 (4)	3.7 (40)
Unclassified/Other	6 (4)	9.3 (99)

**Smoking status % (n)**		
Never Smoked	85 (56)	77.6 (836)
Current Smoker	9 (6)	7.7 (83)
Ex-smoker	6 (4)	14.7 (158)

**PTB risk factors % (n)**		
Previous PTB	37.8 (25)	55.8 (601)
Previous MTL	62.2 (41)	44.2 (476)
Previous cervical surgery	15 (10)	9.3 (101)
Uterine abnormality	7.5 (5)	4.8 (52)

**Gestation at previous preterm event:**		
PTB weeks, median (IQR)	24	32
MTL weeks, median (IQR)	20	20

**Fig. 2:**
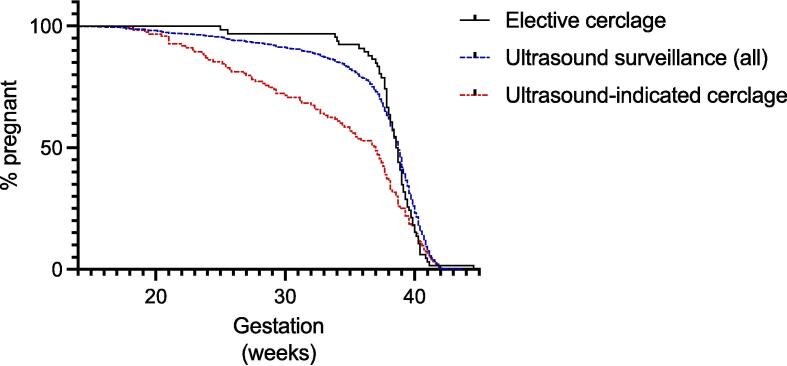
Kaplan Meier survival analysis comparing elective cerclage, ultrasound surveillance and ultrasound-indicated cerclage.

Preterm birth rates were higher in the ultrasound surveillance group, 26% of women delivered before 37 weeks’ gestation, two-times more than in the elective cerclage group (OR 2.3 [CI 1.1–4.5]) (Table 2, Fig. 2).

**Table 2:** Gestation outcomes in women with elective cerclage vs ultrasound surveillance (including those that require an ultrasound-indicated cerclage.

	Elective cerclage (n = 66)	Ultrasound surveillance all (n = 1076)	p Value	Odds Ratio
Gestational age at delivery, mean weeks^+days^ (SD)	38^+1^(2^+6^)	37^+1^(4^+6^)	0.99	

PTB < 37 weeks’ % (n)	13.6 (9)	26.4 (284)	0.02	2.3 (1.1–4.5)
PTB < 32 weeks’ % (n)	3 (2)	10.5 (113)	0.05	3.8 (1–15.8)
PTB < 24 weeks’ % (n)	0 (0)	3.7 (43)	0.1	0

Cerclage inserted, % (n)				
	100 (66)	11.4 (123)		

Average gestation at cerclage (if received) weeks^+days^, mean (SD)	14^+6^ (3^+2^)	18^+2^ (2^+4^)

We then analysed the US-surveillance group, comparing those that had ultrasound surveillance without requiring cerclage with those that had an ultrasound-indicated cerclage (Table 3). As expected, there was a higher chance of preterm birth in the US-indicated cerclage group as these women developed a short cervix (23.3% in ultrasound surveillance only group vs 50.4% in the ultrasound-indicated cerclage group) (Table 3, Fig. 2).

**Table 3:** Gestation outcomes in women with ultrasound surveillance and no subsequent cerclage compared with ultrasound surveillance with cerclage.

	Ultrasound surveillance; no cerclage (n = 953)	Ultrasound surveillance; ultrasound-indicated cerclage (n = 123)	
Gestational age at delivery, weeks^+days^ (mean, SD)	37^+5^ (4^+3^)	33^+6^ (6^+5^)	

PTB < 37 weeks % (n)	23.3 (222)	50.4 (62)	
PTB < 32 weeks % (n)	7.8 (74)	31.7 (39)	
PTB < 24 weeks % (n)	2.8 (27)	13.0 (16)	

Livebirth	97.2 (927)	88.6 (109)	

We then analysed those women that had an ultrasound-indicated cerclage and compared the women that delivered preterm before 37 weeks’ gestation with those that delivered term (Table 4). Cervical length at time of cerclage insertion was significantly shorter in the preterm group, than in the term group (14.5 vs 20.5 mm, p = 0.0024), although the cervicovaginal quantitative fetal fibronectin level was similar (189.5 ng/mL in the preterm group vs. 156 ng/mL in the term group, p = 0.32). The proportion of women who had with an emergency rescue cerclage was higher in the preterm group, although this was not significant (45.9% in the preterm group vs 35.5% in the term group, p = 0.27). Of those women that delivered preterm, over a quarter of them delivered before 24 weeks’ gestation (26.23%). Looking specifically at the type of cerclage inserted, there was a significantly higher proportion of Shirodkar cerclages inserted (rather than McDonald cerclage which does not require bladder mobilisation) in the preterm group (6.56% vs 0% in the term group, p = 0.0194).

**Table 4:** Characteristics in women with who delivered preterm (<37 weeks’ gestation) compared with term following an ultrasound-indicated cerclage.

	US-indicated cerclage; preterm (n = 61)	US-indicated cerclage; term (n = 62)	P value	
**Cerclage subtype (%)**				
Low (McDonald’s)	93.44 (n = 57)	100	0.0194	
High (Shirodkar)	6.56 (n = 4)	0		

**Cervical length before cerclage insertion mm median (IQR)**	14.5 (6.75–21)	20.5 (14.75–23)	0.0024	

**FFN before cerclage insertion ng/mL mean (SD)**	189.5 (193.1)	156 (171.2)	0.32	

**Proportion of rescue stitch (%)**	45.90 (n = 28)	35.48 (n = 22)	0.2735	

**Average GA at delivery (weeks^+days^)**	28^+3^	39^+1^	<0.0001	
**PTB < 37 weeks (%)**	100	0		
**PTB < 32 weeks (%)**	63.93 (n = 39)	0		
**PTB < 24 weeks (%)**	26.23 (n = 16)	0		

**Average gestation at intervention (weeks^+days^)**	19^+1^ (n = 60/61)	19^+3^ (n = 60/62)	0.5223	

## Discussion

In this study, we show that in the women with a prior preterm event, routine transvaginal ultrasound surveillance is associated with 2x higher chance of preterm birth below 37 weeks, than in those women undergoing elective cerclage. However, there is no significant difference between the mean gestational age at delivery in the 2 groups. The majority of women in the ultrasound surveillance group do not require an ultrasound-indicated cerclage, thus preventing an invasive procedure in nearly 90%. However, those women that do have a cerclage are at very high risk of preterm birth, > 50% of these women delivered before 37 weeks’ gestation, so they should be monitored closely [9].

All women included in this study had a prior preterm birth or mid-trimester loss and there was no difference in the rates of additional risk factors for preterm birth between the groups. Women in which elective cerclage were performed were more likely to have experienced a prior mid-trimester loss and it is likely that clinicians inserted an elective cerclage due to clinician and patient anxiety over missing early second-trimester cervical shortening with ultrasound surveillance. This data supports prior evidence that cerclage may be more effective in women with mid-trimester loss as a risk factor [10], [11].

As expected, women with a shorter cervical length at time of ultrasound-indicated cerclage insertion were more likely to deliver preterm, although cervicovaginal fetal fibronectin level was similar between women who delivered preterm and term. There was also a higher proportion of rescue emergency cerclages (indicated when the cervix has opened) in the group that delivered preterm, compared with the women that delivered term (45.9 vs 35.48). This would be consistent with data showing that rescue stitches are only estimated to delay birth by approximately 34 days [12]. There were also more Shirodkar cerclages performed in the preterm group, suggesting a more technically difficult cerclage insertion as well as shorter cervix at time of insertion. Although other studies have shown either no significant differences or a reduction in PTB rates with Shirodkar cerclage compared with McDonald cerclage [13], [14]. Furthermore, the gestation at the time of insertion did not impact on the likelihood of delivering preterm and this was consistent with other retrospective cohort studies [15].

The main strength of this study is a relatively large cohort, all from one centre, resulting in consistent surgical techniques. However, there is still need for prospective randomised studies looking at history and ultrasound surveillance in the cohort of women with one prior preterm event. Due to limited evidence, the RCOG guidance currently recommends that history-indicated cerclage is only indicated following 3 prior preterm events [4]. In view of this data, it would be important to understand the effectiveness of history-indicated cerclage in women following one prior preterm event so that we can better guide and inform clinicians and patients.

One limitation of this study was that we only included women with a prior preterm birth or late miscarriage to make the groups more comparable, however it was difficult to control for other risk factors such as a prior full dilatation caesarean section which is associated with recurrent preterm losses [16], [17]. Furthermore, the data was collected over a ten year period so clinical care and management, specifically ultrasound surveillance and cerclage technique, may have improved over this time period.

## Conclusion

In conclusion, this study shows that in women with one prior preterm event, both history-indicated cerclage and ultrasound surveillance are appropriate management options. However, there does appear to be less preterm births in women with a history-indicated cerclage, although this may reflect the mechanism of their preterm birth phenotype as more of these women had mid-trimester losses. The majority of women undergoing ultrasound surveillance did not require a cerclage and so avoided the potential perioperative complications of cerclage insertion. However, those that did require an ultrasound-indicated cerclage were at high risk of preterm birth so should be followed up closely to enable adequate preterm birth preparation such as antenatal corticosteroids and magnesium sulphate. Unsurprisingly, we show that a shorter cervical length at time of cerclage insertion was associated with more preterm births/mid-trimester losses. Further prospective studies assessing comparing history indicated cerclage and US surveillance in women with a prior preterm event are necessary.

## Declaration of Competing Interest

The authors declare that they have no known competing financial interests or personal relationships that could have appeared to influence the work reported in this paper.
